# Ultrasound-Guided Lower Leg Muscle Compressibility Before and After a Standard Treadmill Test in Healthy Volunteers

**DOI:** 10.1093/milmed/usaf138

**Published:** 2025-09-16

**Authors:** Kay van Heeswijk, Bert van Essen, Loes Janssen, Michiel Winkes, Marc Scheltinga

**Affiliations:** Department of Surgery, Máxima Medical Center, P.O. Box 7777, Veldhoven 5500 MB, The Netherlands; Department of Sports Medicine, Máxima Medical Center, P.O. Box 7777, Veldhoven 5500 MB, The Netherlands; Department of Surgery, Máxima Medical Center, P.O. Box 7777, Veldhoven 5500 MB, The Netherlands; Department of Surgery, VieCuri Medical Center, P.O. Box 1926, Venlo 5900 BX, The Netherlands; Department of Surgery, Máxima Medical Center, P.O. Box 7777, Veldhoven 5500 MB, The Netherlands

## Abstract

**Introduction:**

Military personnel on duty or engaged in sports activities may experience exercise-induced lower leg pain (ELP). If history and physical examination suggest chronic exertional compartment syndrome (CECS) and conservative treatments prove ineffective, a muscle compartment pressure measurement is advised. However, this test is invasive with suboptimal test characteristics. Aim of this study is to determine whether a novel noninvasive soft tissue compressibility analysis using ultrasound can detect changes in anterior tibial muscle (ATM) compartment thickness and compressibility following a standardized treadmill test.

**Methods:**

Healthy volunteers without ELP underwent serial ultrasound measurements of the ATM during rest and after treadmill walking (5.5 km/h, slope 15%). Compartment thickness, defined as the distance between the superficial fascia and two different deep landmarks (interosseous membrane (IM), or transition zone IM to tibia (TZIT)) was measured with 10 mmHg (d10) and 80 mmHg (d80) external probe pressure. Compartment compressibility was calculated as a ratio (d10-d80)/d10*100%.

**Results:**

Healthy volunteers (*n* = 70, males *n* = 28, age 19-72 years) were included (IM *n* = 35; TZIT *n* = 35). Compartment *thickness* (d80) during rest, 1- and 5-minutes post-exercise with IM as internal landmark was 25.9mm (±2.7), 27.0mm (±3.1; *p* < .01), and 26.5mm (±2.7; *p* < .01), respectively. Using TZIT as landmark, these values were 22.2 mm (±3.1), 23.8 mm (±3.7; *p* <  .01) and 23.0 mm (±3.7; *p* < .07). Compartment *compressibility* with the IM as landmark during rest was 9.5% (±2.5) and did not significantly change post-exercise (*p *= 0.45). Using TZIT, compressibility was 15.0% (±4.2) at rest, and decreased to 13.1% (±3.8; *p* =  .02) and 14.1% (±4.5; *p* =  .21) 1- and 5 min post-exercise, respectively.

**Conclusions:**

Soft tissue compressibility analysis using ultrasound successfully detected changes in ATM compartment thickness following a standard treadmill test. Changes in muscle compressibility depended on type of internal anatomical landmark used. Future research focuses on lower leg muscle compressibility in individuals with exercise-induced leg pain.

## INTRODUCTION

Physically active military personnel may develop exercise-induced lower leg pain (ELP), which is occasionally caused by a chronic exertional compartment syndrome (CECS).^1^ Approximately thousand new cases are diagnosed annually in the U.S. military.^2^ It is believed that CECS is caused by an abnormal rise in muscle compartment pressure and most commonly affects the lower leg’s anterior compartment (ant-CECS).^3^ Symptoms are pain and tightness during and after exertion.^4^ If conservative treatments including gait retraining are ineffective, a intracompartmental pressure measurement (ICPM) is advised.^5^ However, this invasive technique is uncomfortable for patients and its diagnostic characteristics are suboptimal.^6–8^ Moreover, the level of pain is not necessarily correlated with compartment pressure values.^9^ Therefore, there is a need for noninvasive diagnostic tools with optimal sensitivity and specificity.

A range of diagnostic tools is currently being explored for ant-CECS.^10,11^ Of these tools, only a limited number of studies have investigated the use of ultrasound in CECS.^12–16^ The lack of standardization of external pressure applied by the ultrasound probe is concerning, as previous research has demonstrated that compartment thickness measurements are directly influenced by the amount of external force applied by the transducer probe.^17,18^ Correcting for this, a new ultrasound technique using soft tissue compressibility analysis was found to correctly diagnose acute compartment syndrome (ACS).^19–22^

Recently, ultrasound-based muscle compartment thickness measurements at 10 mmHg (d10) and 80 mmHg (d80) external probe pressure were performed in healthy volunteers at rest.^17,18^ The aim of the present study is to investigate whether soft tissue compressibility analysis can detect changes in muscle compartment thickness and compressibility following a standardized treadmill test in a diverse group of asymptomatic, healthy volunteers.

## METHODS

### General Study Information

The study was conducted at Máxima Medical Center (MMC), Veldhoven, the Netherlands between February 2023 and February 2024. This hospital hosts a center of expertise on ELP syndromes. The procedures of the study complied with the Declaration of Helsinki (1964) and its amendments. The study protocol was approved by MMC’s independent medical ethical testing committee (NL82601.015.22). The trial was registered at clinicaltrials.gov under reference number NCT05720182. Safety of the muscle compressibility device was confirmed in recent studies.^18–20,17,22^ The device is FDA-cleared and meets MIL-STD 810 standards.

### Participants

Study participants were recruited among hospital colleagues and relatives of the investigators via public talks and advertisements. Eligibility criteria included age older than 18 years and the absence of muscle disorders or arteriovenous diseases. ELP-related complaints such as pain, tightness, and cramps as well as prior lower leg traumas were excluded based on a completed questionnaire routinely used during the intake of ELP patients in our department.^24,25^ Participants were also excluded from analysis if they were unable to complete the exercise protocol.

### Muscle Compressibility Device

The device combines ultrasound with a pressure measurement modality (CPMX1, Compremium AG, Switzerland; www.compremium.ch) and has been described in detail previously.^17^ To summarize, the probe is manually positioned by the investigator on a predefined external landmark of the anterior portion of the lower leg and is directed towards one of the two currently used internal landmarks. Time-synchronized ultrasound images are automatically obtained at 10 mmHg and 80 mmHg external ultrasound probe pressure. The integrated software (1.0.2-alpha.0.14) displays compartment thickness at both pressures (d10, d80; millimeters) and calculates muscle compressibility using the formula (d10-d80)/d10*100%.

### Protocol of Exercise Measurements

All 70 participants underwent measurement sessions by a single observer. Participants wore shorts and sports or comfortable shoes. The examination room was maintained at approximately 20 °C. Before measurements, participants rested in a supine position for at least 5 min on an examination bench. Both legs were supported at the knee level with a triangular pillow and at the ankle level.^23^ This position is also standard in ELP patients undergoing ICP measurements. However, this position differs from the protocol of the manufacturer advising leg support at the medial side with a cushion. The side of measurement (left or right leg) was randomized.

The first group of 35 participants underwent measurements using the Transition Zone Interosseous membrane to Tibial bone (TZIT) as the internal landmark, per the manufacturer’s guidelines. Initially, the distance (in centimeters) between fibular head and lateral malleolus was measured. At 40% of this distance approximately 2 cm lateral to the anterior tibial crest, the ultrasound probe position was temporarily marked with a skin marker.^15,17^ Once the TZIT was clearly visible, a definitive skin marking was applied. Muscle compressibility was measured four times over a 2-3 min period. Participants then completed a standardized 5 min treadmill exercise test (5.5 km/h, 15% slope). Post-exercise measurements were taken at approximately 30 s, and 1, 2, 3, and 5 min after the treadmill test. During the treadmill exercise, participants verbally reported the maximal pain score at the anterior compartment (NRS, range 0-10). After a 5 minute rest period, the exercise protocol was repeated on the contralateral leg.

Given that previous studies demonstrated significant differences in compressibility and compartment thickness using TZIT compared to an alternative landmark termed the interosseous membrane (IM),^17^ a second group of 35 volunteers underwent the same protocol using the middle of the IM as internal landmark.

### Outcomes and Confounders

The primary outcome was the difference in muscle compartment thickness and muscle compressibility at two external probe pressures (10 mmHg and 80 mmHg) before and after a standard treadmill test. Secondary outcome included *intra*-observer reliability for measurements at rest. Additional confounding variables analyzed were gender (male, female), age (<30 years, 30-49 years, ≥50 years), and BMI (<25 kg/m^2^, ≥25 kg/m^2^).

### Sample Size Calculation

Sample size was determined using G*Power 3.1^26^ based on the primary outcome. Calculations were performed according to published data.^18,20,23^ A dependent *t*-test with expected effect size of 0.4, an α of 0.05, power (β-1) of 0.90 resulted in a required sample size of n = 68 legs. Accounting for a 1% drop-out or faulty measurements, 35 participants per internal landmark group were required.

### Statistical Analysis

Data were analyzed using SPSS Software (IBM Corp., Armonk, NY) version 29. Data variables were checked for normality using kurtosis (between −1.0 and 1.0), skewness (between −1.0 and 1.0) and Shapiro-Wilk test. If normal, they were depicted as mean (± standard deviation; SD). If not, they were depicted as median (interquartile range; IQR). For comparison of rest measurements between subgroups (based on gender, age, and BMI) and between landmarks, independent *t*-tests were used for normally distributed data, and Mann-Whitney *U*-tests for non-normal distributed data. Each post exercise time point (30 s, 1 min, 2 min, 3 min, and 5 min post exercise) was separately compared to the mean of the four rest measurements using dependent *t*-tests (normal distribution) or Wilcoxon Signed Rank tests (non-normal distribution). A Bonferroni-correction, used to reduce the risk of false-positive results, was used for multiple comparisons. The data were analyzed as grouped for the two internal landmarks (TZIT and IM). A *p*-value < .01 was considered significant for the exercise measurements. A measurement was excluded if the compressibility value was negative assuming it was an incorrect measurement.

The *intra-observer* reliability for d10, d80 and compressibility was assessed using the intraclass correlation coefficient (ICC) with 95%-confidence interval. The data were based on averages of four consecutive rest measurements. Reliability was analyzed using a two-way random, single-observer, absolute agreement model. An ICC <0.5 was considered as poor, 0.5-0.7 as moderate, 0.7-0.9 as good and >0.9 as excellent according to Koo et al.^26^

## RESULTS

All 70 study participants (28 males, age range 19-72 years, BMI range 18-32) provided verbal and written consent and completed the measurements without reporting any discomfort, except for fatigue after the treadmill test in some individuals. One participant was excluded as unable to complete the study protocol due of exhaustion. As per protocol, two negative compressibility measurements of a total of 630 measurements were considered faulty and were excluded. Participant demographics are summarized in **Table 1**.

**Table 1.** Demographics of Two Groups Undergoing Soft Tissue Compressibility Analysis of the Lower Leg Muscle using the IM or TZIT Internal Landmark Protocol.

	IM (*n* = 35)	TZIT (*n* = 35)	*p*-value
Sex (males | females)	14 | 21	14 | 21	.89
Age in years (median; IQR)	32 ; 29	40 ; 32	.14
<30 (*n*)	16	12	
30-50 (*n*)	9	7	
>50 (*n*)	10	16	
BMI in kg/m^2^ (mean ± SD)	24.1 ± 3.6	24.1 ± 3.5	.82
<25 (*n*)	22	22	
≥25 (*n*)	13	13	

### Rest Measurements

Compartment thickness and compressibility values at rest are presented in **Table 2**. No significant differences were observed between left and right legs. Thickness values at d10 and d80 using the IM landmark was significantly higher compared to TZIT for BMI groups, sexes and the <30 years age group (*p* < .01). In contrast, no differences were observed for age group “30-50 years” (*p* = .32) and “>50 years” (*p* = .13). Measurements of thickness demonstrated “excellent” ICC, while compressibility showed “good” reliability.

**Table 2.** Lower Leg Anterior Muscle Compartment Thickness and Compressibility During Rest (*n* = 70 Healthy Volunteers, Average of Four Measurements)

		Muscle thickness at d10 (mm)	Muscle thickness at d80 (mm)	Compressibility (%)
IM	All measurements (Mean ± SD)	28.7 (3.1)*	25.9 (2.7)*	9.5 (2.5)*
	Male (Median; IQR)	31.9 (4.5)	28.6 (3.8)	9.7 (2.5)
	Female (Median; IQR)	27.6 (3.6)**	25.2 (3.4)**	9.6 (5.1)
	<30 Years (Median; IQR)	28.5 (4.9)	25.7 (4.1)	10.0 (2.8)
	30–50 Years (Median; IQR)	28.1 (5.6)	25.8 (4.8)	10.4 (4.0)
	>50 Years (Median; IQR)	28.3 (5.5)	26.4 (5.1)	8.5 (4.4)
	BMI <25 (Median; IQR)	28.1 (4.7)	25.3 (3.8)	9.0 (3.5)
	BMI ≥25 (Median; IQR)	29.7 (5.2)	27.1 (4.2)	9.7 (3.6)
	ICC (95%-CI)	0.99 (0.98-1.0)	99 (0.98-1.0)	0.77 (0.65-0.87)
TZIT	All measurements (Mean ± SD)	26.1 (3.1)	22.2 (3.1)	15.0 (4.2)
	Male (Median; IQR)	28.6 (1.9)	24.7 (2.4)	13.1 (6.9)
	Female (Median; IQR)	25.2 (4.8)**	21.0 (4.2)**	15.0 (6.4)
	<30 Years (Median; IQR)	27.4 (4.6)	23.9 (4.5)	13.1 (3.1)
	30-50 Years (Median; IQR)	25.3 (3.8)	21.3 (5.3)	16.9 (5.7)
	>50 Years (Median; IQR)	26.8 (4.7)	22.7 (4.5)	15.6 (6.1)
	BMI <25 (Median; IQR)	26.2 (4.9)	22.1 (5.0)	13.8 (5.3)
	BMI ≥25 (Median; IQR)	27.3 (3.7)	23.6 (4.4)	16.2 (6.6)
	ICC (95%-CI)	0.94 (0.90-0.97)	0.93 (0.88-0.96)	0.78 (0.67-0.87)

Two internal landmarks: Interosseous Membrane (IM), Transition Zone IM to Tibial bone (TZIT). d10, compartment thickness at 10 mmHg external ultrasound probe pressure; d80, compartment thickness at 80 mmHg external pressure; Compressibility (d10-d80)/d10*100%.

SD Standard Deviation; IQR Inter Quartile Range; BMI Body Mass Index; ICC intraclass correlation.

* , Independent *t*-test *p* <0.05 versus TZIT.

** , Mann-Whitney U *p* <0.05 versus male.

### Post-exercise Measurements

**Figure 1.**
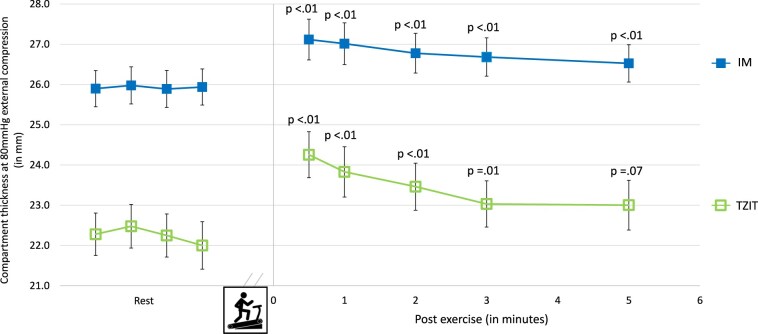
Anterior tibial muscle compartment thickness at 80 mmHg external compression in rest and after a standardized treadmill exercise in 70 healthy volunteers using two internal landmarks: Interosseous Membrane (IM, closed square) and Transition Zone Interosseous membrane to Tibial bone (TZIT; open square). Error bars represent ± SEM. P-values compare post-exercise values to rest averages.

During the treadmill test, participants reported a median anterior compartment pain score of 0 (NRS, range 0-4 on a 0-10 scale). The first post-exercise measurement occurred 33 seconds (±6) after completion of the treadmill test. Compartment thickness values at 80 mmHg external probe pressure are shown in Figure 1 (measurements at 10 mmHg followed similar patterns and are therefore not depicted). Using the IM landmark, compartment thickness remained significantly higher at all 5 time points post exercise compared to rest values. Using TZIT as internal landmark, values up to 3 minutes post exercise were significantly different from rest values.

Following the 5 min rest period after the treadmill test, thickness values remained elevated, indicating incomplete normalization. Consequently, data of the second treadmill session were not analyzed.

**Figure 2.**
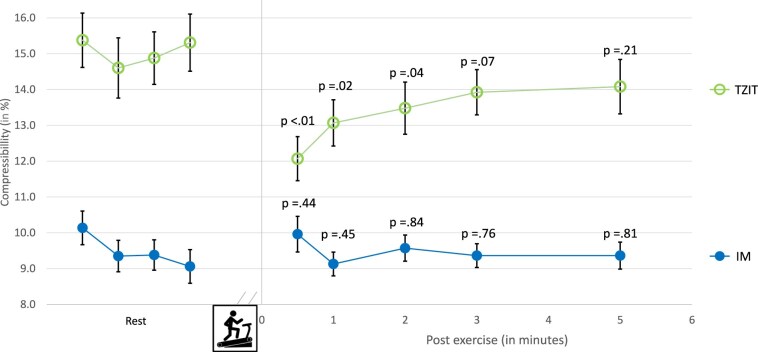
Anterior tibial muscle compartment compressibility (relative difference between compartment thickness at 10 mmHg and 80 mmHg external exerted compression) in rest and after a standardized treadmill exercise in 70 healthy volunteers using two internal landmarks: Interosseous Membrane (IM, closed circle) and Transition Zone Interosseous membrane to Tibial bone (TZIT; open circle). Error bars represent ± SEM. *P*-values compare post-exercise values to rest averages.

Changes in muscle compressibility are presented in Figure 2. With the TZIT landmark, compressibility values returned to baseline within 1-min post-exercise. Compressibility values obtained with the IM landmark showed no significant changes. No gender differences in compressibility were observed at any time point.

## DISCUSSION

If exercise induced lower leg pain (ELP) is likely caused by chronic exertional compartment syndrome (CECS), an intracompartmental pressure (ICP) measurement is typically indicated. However, this diagnostic tool is invasive and lacks precision. Recent studies suggest that soft tissue compressibility analysis using ultrasound may play a role in diagnosing CECS.^4,10^ The present study investigated whether this tool can detect changes in anterior tibial muscle (ATM) compartment thickness and compressibility following a standardized treadmill test in asymptomatic healthy volunteers. The results indicate that changes in muscle thickness and compressibility can be detected and depend on type of internal anatomical landmark. Future studies should focus on testing this technique in ELP patients suspected of having CECS.

It is hypothesized that muscle compartment thickness may increase immediately after exercise because of increased blood flow. However this change in thickness might be less noticeable because of less resistance longitudinally in the ATM compared to the rigidity cross-sectional. For instance, Brahim et al.^16^ reported a 10% increase in ATM thickness using a ultrasound technique, dependent on the intensity of the exercise protocol. Birtles et al.^13^ reported an 8% increase after a 20 minute isometric exercise, also using ultrasound. However, a second study of Birtles et al.^12^ failed to confirm these changes. Comparable studies using other imaging modalities to assess compartment thickness changes after exercise were not identified in the literature. All of the aforementioned ultrasound studies used the IM as the internal landmark but lacked standardized probe pressure. Recent evidence has demonstrated that ATM compartment thickness is significantly influenced by the pressure exerted by the ultrasound probe.^17,18^ The present study showed that a standard treadmill test increased ATM thickness by 4-10%, depending on type of internal landmark. Notably, ATM thickness did not normalize after 5 minutes of rest following the standardized treadmill test. These data suggest that individuals should rest for at least 5 min before undergoing a baseline soft tissue compressibility analysis using ultrasound.

While the effect of a standard treadmill test on ATM thickness in healthy volunteers is predictable, the effect on compartment compressibility is not. A literature search did not identify studies examining muscle compressibility after exercise in healthy populations. In asymptomatic individuals, it could be hypothesized that compressibility would remain unchanged. However, the present study found that compressibility decreased by 20% within 30 s of exercise cessation when using the TZIT landmark and normalized in the min thereafter. Conversely, compressibility using the IM remained unaltered during 5 min of observation following the treadmill test. Variability in compressibility values with the IM landmark was lower compared to TZIT landmark. These findings suggest that changes in ATM compressibility are influenced by probe location and orientation, potentially reflecting differences in arterial and venous circulation in various portions of the ATM. The IM landmark may be preferred as a result of lower variability in compressibility values. Furthermore, it is questionable whether individuals without any leg discomfort during the treadmill test would exhibit substantial alterations in ATM compressibility.

In general, males have greater muscle mass than females, which could influence muscle compressibility. Previous studies have not examined the relationship between sex and compressibility. Birtles et al.^13^ mentioned that their values are in a normal range for females, but did not elaborate on differences between males and females. The present study found that a male ATM muscle was approximately 15% thicker compared to a female ATM, independent of the internal landmark. However, muscle compressibility was similar between sexes for the IM landmark (male 9.7% (2.5) vs female 9.6% (5.1), *p* = .15) and TZIT landmark (male 13.1% (6.9) vs. female 15.0% (6.4), *p* = .34). The relationship between thickness of subcutaneous fat layer and muscle compressibility remains unknown. These findings suggest that compressibility may be unrelated to both compartment thickness and subcutaneous fat layer. Future studies should investigate the potential association between fat layer thickness and ATM compressibility.

This study has limitations including a relatively small sample size, which precluded stratification based on demographics. To minimize bias, all measurements were performed by a single observer. The study also found that a baseline rest state was not achieved in all participants after 5 min of rest, potentially resulting in falsely elevated muscle thickness measurements. Consequently, data obtained after the second exercise session were excluded. Another limitation is that the first 35 individuals were assessed using the TZIT landmark, while the IM landmark was used for the second group of 35 individuals.

## CONCLUSIONS

Soft tissue compressibility analysis using an ultrasound technique can detect changes in anterior tibial muscle compartment thickness following a standardized treadmill test in healthy volunteers. Changes in muscle compressibility were dependent on the type of internal anatomical landmark. Future research should focus on soft tissue compressibility analysis in patients with exercise-induced leg pain, including CECS.

## INSTITUTIONAL CLEARANCE

Institutional approval granted by the local approval body “Commissie Lokale Uitvoerbaarheid” (022-MMC-098).

## References

[1] Chandwani D, Varacallo M. *Exertional Compartment Syndrome*. StatPearls Publishing; 2023. 31335004

[2] Waterman RB, Liu J, Newcomb R, et al. Risk factors for chronic exertional compartment syndrome in a physically active military population. *Am. J. Sports Med*. 2013;41(11):2545–9.doi:doi: 10.1177/0363546513497922 23911700

[3] Winkes MB, Bloo H, Hoogeveen AR, and Scheltinga M: Het chronisch inspanningsgebonden compartimentsyndroom van het onderbeen. *Physios*. 2018;38(4):11-19.

[4] Tarabishi MM, Almigdad A, Almonaie S, Farr S, Mansfield C: Chronic exertional compartment syndrome in athletes: an overview of the current literature. *Cureus*. 2023;15(10):e47797. 10.7759/cureus.47797 PMC10676709 38022185

[5] Dean RS, Farley KX, Waterman BR, Guettler J, Bicos J. Chronic exertional compartment syndrome is frequently diagnosed through static compartment pressure measurements and managed with fasciotomy: A systematic review. *J ISAKOS*. 2024;9(1):71–8.doi:doi: 10.1016/j.jisako.2023.09.005 37778507

[6] Large TM, Agel J, Holtzman DJ, Benirschke SK, Krieg JC. Interobserver variability in the measurement of lower leg compartment pressures. *J Orthop Trauma*. 2015;29(7):316–21.doi: 10.1097/BOT.0000000000000317 25756911

[7] Pasic N, Bryant D, Willits K, and Whitehead D. Assessing outcomes in individuals undergoing fasciotomy for chronic exertional compartment syndrome of the leg. *Elsevier BV*. 2015;31(4):707. 10.1016/j.arthro.2014.10.018 25543245

[8] Nakhostine M, Styf JR, van Leuven S, Hargens AR, Gershuni DH. Intramuscular pressure varies with depth: the tibialis anterior muscle studied in 12 volunteers. *Acta Orthop Scand*. 1993;64(3):377–81.doi: 10.3109/17453679308993649 8322604

[9] Zimmermann WO, Ligthert E, Helmhout PH, et al Intracompartmental pressure measurements in 501 service members with exercise-related leg pain. *Transl. J. Am. Coll. Sports Med*. 2018;3(14):107–12.doi: 10.1249/TJX.0000000000000065

[10] der Kraats A, Winkes M, Janzing HMJ, Eijkelenboom RPR, de Koning MTG. Review of reliable and valid noninvasive tools for the diagnosis of chronic exertional compartment syndrome. *Orthop. J. Sports Med*. 2023;11(1):23259671221145151.doi:doi: 10.1177/23259671221145151 PMC9841863 36655016

[11] Ritchie ED, Vogels S, Van Dongen TTCF, et al Systematic review of innovative diagnostic tests for chronic exertional compartment syndrome. *Georg Thieme Verlag KG*. 2022;44(01):20. 10.1055/a-1866-5957 PMC9815949 35649437

[12] Birtles DB, Rayson MP, Jones DA, et al. Effect of eccentric exercise on patients with chronic exertional compartment syndrome. *Eur. J. Appl. Physiol*. 2003;88(6):565–71.doi: 10.1007/s00421-002-0740-z 12560956

[13] Birtles DB, Minden D, Wickes SJ, et al Chronic exertional compartment syndrome: muscle changes with isometric exercise. *Med Sci Sports Exercise*. 2002;34(12):1900–6.doi: 10.1097/00005768-200212000-00007 12471294

[14] Gershuni DH, Gosink BB, Hargens AR, et al Ultrasound evaluation of the anterior musculofascial compartment of the leg following exercise. *Clin Orthop Relat Res*. 1982;167(6):185–90.doi: 10.1097/00003086-198207000-00028 7094462

[15] Rajasekaran S, Beavis C, Aly A-R, Leswick D. The utility of ultrasound in detecting anterior compartment thickness changes in chronic exertional compartment syndrome: a pilot study. *Clinical J Sport Med*. 2013;23(4):305–11.doi: 10.1097/JSM.0b013e3182856046 23558330

[16] Brahim F, Zaccardelli W. Ultrasound measurement of the anterior leg compartment. *Am. J. Sports Med*. 1986;14(4):300–2.doi: 10.1177/036354658601400410 3524281

[17] van Heeswijk K, Janssen L, Heijmans MH, and Scheltinga MRM. Ultrasound guided compressibility of the lower leg anterior tibial muscle compartment: a feasibility study. *Phys Sportsmed*. 2024;52(6):1–7. 38600863 10.1080/00913847.2024.2340421

[18] Anwander H, Büchel L, Krause F, Siebenrock K, Schmid T. Tibial anterior compartment compressibility in healthy subject, measured using compression sonography. *Injury*. 2022;53(2):719–23.doi: 10.1016/j.injury.2021.12.014 34963511

[19] Sellei RM, Beckers A, Kobbe P, et al Non-invasive assessment of muscle compartment elasticity by pressure-related ultrasound in pediatric trauma: a prospective clinical study in 25 cases of forearm shaft fractures. *Eur. J. Med. Res*. 2023;28(1):296.doi: 10.1186/s40001-023-01232-1 PMC10463760 37626380

[20] Sellei RM, Wollnitz J, Reinhardt N, et al Non-invasive measurement of muscle compartment elasticity in lower limbs to determine acute compartment syndrome: Clinical results with pressure related ultrasound. *Injury*. 2020;51(2):301–6.doi: 10.1016/j.injury.2019.11.027 31784057

[21] Herring MJ, Donohoe E, and Marmor MT. A novel non-invasive method for the detection of elevated intra-compartmental pressures of the leg. *J. Vis. Exp*. 2019;147(5):e59887. 10.3791/59887 31205299

[22] Marmor MT, Barker JP, Matz J, Donohoe E, Herring MJ. A dual-sensor ultrasound based method for detecting elevated muscle compartment pressures: A prospective clinical pilot study. *Injury*. 2021;52(8):2166–72.doi:doi: 10.1016/j.injury.2021.02.054 33640161

[23] de Bruijn JA, van Zantvoort APM, van Klaveren D, et al Factors predicting lower leg chronic exertional compartment syndrome in a large population. *Int J Sports Sci Med*. 2018;40(01):58–66. 10.1055/s-0043-119225 29126337

[24] van Zantvoort APM, Hundscheid HPH, de Bruijn JA, et al. Isolated Lateral Chronic Exertional Compartment Syndrome of the Leg: A New Entity?. *Orthop J Sports Med*. 2019;7(12):2325967119890105.doi:doi: 10.1177/2325967119890105 PMC6931149 31903402

[25] Faul F, Erdfelder E, Buchner A, Lang A-G. *Statistical Power Analyses Using G*Power 3.1: Tests for Correlation and Regression Analyses*. Springer Science and Business Media LLC; 2009. 1149. 10.3758/BRM.41.4.1149 19897823

[26] Koo TK, Li MY. A guideline of selecting and reporting intraclass correlation coefficients for reliability research. *J. Chiropr. Med*. 2016;15(2):155–63.doi:doi: 10.1016/j.jcm.2016.02.012 27330520 PMC4913118

